# SOD-Mimic Cu(II) Dimeric Complexes Involving Kinetin and Its Derivative: Preparation and Characterization

**DOI:** 10.1155/2012/704329

**Published:** 2012-08-27

**Authors:** Radka Novotná, Zdeněk Trávníček, Radovan Herchel

**Affiliations:** Department of Inorganic Chemistry, Regional Centre of Advanced Technologies and Materials, Faculty of Science, Palacký University, 17. listopadu 12, 771 46 Olomouc, Czech Republic

## Abstract

Two SOD-mimic active dimeric Cu(II) chlorido complexes of the compositions [Cu_2_(**μ**-HL^1^)_4_Cl_2_]Cl_2_ (**1**) and [Cu_2_(**μ**-HL^2^)_2_(**μ**-Cl)_2_(HL^2^)_2_Cl_2_] *·* 4H_2_O (**2**) involving the cosmetologically relevant cytokinin kinetin (N6-furfuryladenine, HL^1^) and its derivative N6-(5-methylfurfuryl)adenine (HL^2^) have been synthesized and characterized by elemental analysis, infrared, and electronic spectroscopy, ESI+ mass spectrometry, conductivity and temperature dependence of magnetic susceptibility measurements, and thermogravimetric (TG) and differential thermal (DTA) analyses. The results of these methods, particularly the temperature dependence of magnetic susceptibility, showed the complexes to be dimeric with a strong antiferromagnetic exchange (*J* = −290 cm^−1^ for complex **1** and *J* = −160 cm^−1^ for **2**). The complexes have been identified as auspicious SOD-mimics, as their antiradical activity evaluated by the *in vitro* SOD-mimic assay resulted in the IC_50_ values equal to 8.13 **μ**M (**1**) and 0.71 **μ**M (**2**).

## 1. Introduction

The metalloenzyme copper, zinc-superoxide dismutase (Cu, Zn-SOD), plays a crucial role in the cell protection against the damage of physiological functions caused by the reactive oxygen species in mammals [1]. The SOD enzyme catalyses the disproportionation of the cytotoxic superoxide radical to oxygen and hydrogen peroxide [2]. The overproduction or destructive effect of superoxide is a basic characteristic of more than 100 diseases, including neurodegenerative [3, 4], diabetes [5], inflammatory or carcinogenetic related processes [6]. The Cu, Zn-SOD enzyme, which is the organism's first line of antioxidant defence, contains a bimetallic active site with zinc(II) and copper(II) bridged by deprotonated imidazole. The presence of the redox active copper atom is essential for the superoxide dismutase mechanism and if zinc is replaced by copper, the dicopper derivative has the same activity as the native enzyme [7]. The unique role of this enzyme in the organism protection has made it a model compound for the design of low molecular antioxidants on the basis of copper complexes mimicking the active site of the metalloenzyme, thus achieving SOD-like activity. Metallotherapeutics based on such SOD-mimics could then be supplemented in the treatment of diseases connected with the oxidative stress. This assumption that low molecular SOD-mimic Cu(II) complexes could have a potential to become future pharmaceuticals has recently been confirmed by results of our laboratory. The dimeric perchlorate Cu(II) complexes [Cu_2_(*μ*-HL^*x*^)_4_(ClO_4_)_2_](ClO_4_)_2_ involving variously benzyl-substituted derivatives of N6-benzyladenine (HL^*x*^), a natural phytohormone, exhibited promising SOD-mimic activity (IC_50_ = 8.67–41.45 *μ*M) [8]. Moreover, the complexes of the same basic composition, however, with the chloride anions instead of perchlorate, showed dramatically increased activity (IC_50_ = 0.687–1.090 *μ*M) and in the case of structurally different [Cu_2_(*μ*-2MeOHL^*x*^)_2_(*μ*-Cl)_2_Cl_2_] even better (IC_50_ = 0.253 *μ*M) than the native bovine Cu, Zn-SOD (0.48 *μ*M); 2MeOHL^*x*^ = N6-(2-methoxybenzyl)adenine [9]. Additionally, the pharmaceutical potential of the latter complexes involving the methoxy-benzyl-derived N6-benzyladenines was confirmed in the antidiabetic activity testing (cytoprotective effect against the alloxan-induced diabetes), in which they also exhibited significant antioxidant activity *in vivo *[9]. The last mentioned promising results motivated us to try to prepare Cu(II) chlorido complexes involving the molecule of kinetin. Kinetin (N6-furfuryladenine) belongs among naturally occurring phytohormones called cytokinins. Apart from its action in plants, it has been shown to possess strong antiageing activity in fruit flies and also human fibroblasts, for which it is presently used in cosmetic pharmaceuticals. As for the suggested mode of kinetin action, various evidence indicates that kinetin can act both as an inhibitor of the radical oxygen species (ROS) formation and as a scavenger of ROS [10]. Taking into consideration these intrinsic antioxidant properties of kinetin and the high antioxidant activity of the above-mentioned Cu chlorido complexes of N6-benzyladenine [9], Cu(II) chlorido complexes involving the molecule of kinetin as a ligand could exhibit high SOD-mimic activity based on the possible synergic effect of the ligand and redox active copper centres.

## 2. Experimental

### 2.1. Materials and Methods

**Figure 1:**
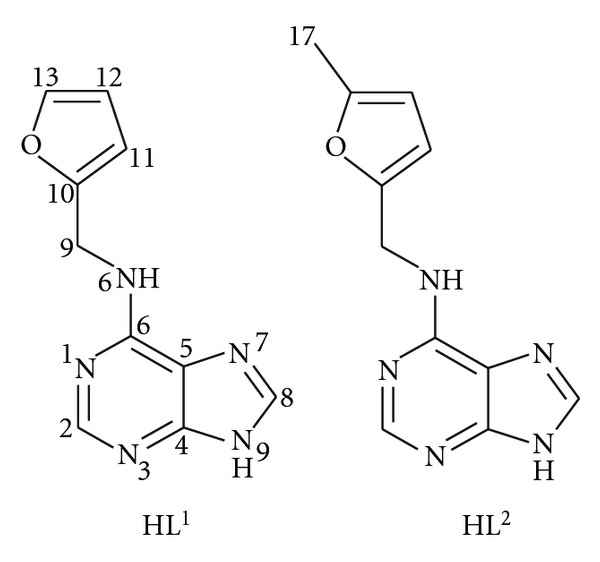
The organic compounds (N6-furfuryladenine, left; N6-(methylfurfuryl)adenine, right) used as ligands in the presented complexes.

Chemicals and solvents were obtained from commercial sources (Sigma-Aldrich Co., Acros Organics Co.) and were used as received. N6-furfuryladenine (HL^1^) and its derivative, N6-(5-methylfurfuryl)adenine (HL^2^), (Figure 1), were synthesized using the modified previously reported procedures [11].

Elemental analyses (C, H, N) were measured on a ThermoScientific Flash 2000 CHNS-O Analyser. Conductivity measurements (10^−3^ M DMF solutions; 25°C) were obtained on a Cond 340i/SET (WTW) conductometer. The copper content was measured by chelatometric titration with murexide as an indicator. After mineralizing the complexes in concentrated HNO_3_, the resulting suspension was dissolved in distilled water. Then a few drops of a buffer (1 M NH_3_) were added and the solution turned dark blue, after which the indicator murexide was added to obtain a brown colour. The resulting solution was titrated with a solution of complexon III until a purple colour. The diffuse reflectance and electronic absorption spectra (10^−3^ M DMF and DMSO solutions of complexes) were measured on a Perkin-Elmer Lambda35 spectrometer (200–1000 nm). FTIR spectra were recorded on a Nexus 670 FT-IR (ThermoNicolet) using the ATR (400–4000 cm^−1^) and Nujol techniques (150–600 cm^−1^). The mass spectra were measured on a LCQ Fleet Ion Mass Trap mass spectrometer (Thermo Scientific). A SQUID magnetometer (Quantum Design) was used for the magnetic susceptibility measurements in the temperature range of 2–300 K with the applied field of 1 T. The diamagnetic corrections were calculated using Pascal constants [12] and the correction for the temperature-independent paramagnetism *χ*_TIP_ = +0.75 m^3^ mol^−1^ per a Cu(II) ion was applied. Thermogravimetric (TG) and differential thermal analyses (DTA) were taken on an Exstar TG/DTA 6200 (Seiko Instruments Inc.) thermal analyzer in a ceramic crucible in dynamic air atmosphere (150 mL min^−1^) up to 1000°C (2.5°C min^−1^ gradient).

### 2.2. SOD-Mimic Activity Testing

The SOD-mimic activity of the complexes **1** and **2** was evaluated by a modified indirect chemical method, which was described previously [13]. The method involves the competitive reaction between the tested compounds and 2,3-bis(2-methoxy-4-nitro-5-sulfophenyl)-2H-tetrazolium-5-carboxanilide sodium salt [the XTT dye] with the saturated DMSO solution of KO_2_. The concentration of orange XTT-formazane, as the product of the reaction of XTT with superoxide, was determined by UV-Vis spectra measurements at 470 nm. The percentage of inhibition of XTT reduction was calculated using the equation, %INH = 100 ∗ (*A*_*b*_− *A*_*s*_)/*A*_*b*_, where *A*_*b*_ (blank) and *A*_*s*_ (sample) are absorbances at 470 nm. The IC_50_ values were then calculated from the linearized dependence of %INH on a logarithm of molar concentration (first order equation). The concentration that caused 50% inhibition of the XTT-formazane formation (IC_50_) was compared to the standard of bovine Cu, Zn-superoxide dismutase.

### 2.3. Syntheses of Cu(II) Complexes

#### 2.3.1. [Cu_2_(*μ*_2_-HL^1^)_4_Cl_2_]Cl_2_ (**1**)

The ligand HL^1^ (1 mmol) was dissolved in 50 mL methanol under reflux and then, CuCl_2_·2H_2_O dissolved in a minimum of warm distilled water was added. The resulting mixture immediately turned into thick dark green suspension, which was then slowly filtered off and washed with cold methanol and diethyl ether and dried under an infrared lamp.

[Cu_2_(*μ*_2_-HL^1^)_4_Cl_2_]Cl_2_ (**1**): yield: 73% [with respect to (wrt) copper], Anal. Calc. for Cu_2_Cl_4_C_40_H_36_N_20_O_4_ (Mr = 1129.8): C, 42.5; H, 3.2; N, 24.8; Cu, 11.2. Found: C, 42.4; H, 3.6; N, 24.5; Cu, 11.6%. Λ_M_ (DMF solution, S cm^2^ mol^−1^): 131.3. FTIR (Nujol, cm^−1^): 278 s (Cu–N), 331 s (Cu–Cl). FTIR (ATR, cm^−1^): 1504 w, 1540 m, 1592 sh (C=C), 1616 s, 1642 s (C=N), 2933 m (C–H)_al_, 3057 w, 3124 m (C−H)_ar_, 3271 m (N–H). *λ*_max⁡_ (solid state, nm): 611. *λ*_max⁡_ (10^−3^ M DMF solution, nm)/*ε* (M^−1^ cm^−1^): 636/97. ESI + MS (m/z): 136, 148, 216, 278, 493, 527, 557, 591.

#### 2.3.2. [Cu_2_(*μ*-HL^2^)_2_(*μ*-Cl)_2_(HL^2^)_2_Cl_2_]·4H_2_O (**2**)

The modification of the previously described procedure established by Mikulski et al. for adenine complexes with divalent 3d metal chlorides was applied [14]. Hydrated copper chloride (1.4 mmol) was dissolved in a mixture of methanol (35 mL) and triethylorthoformate (15 mL) and stirred at 50°C for 1 h. Subsequently, the organic compound HL^2^ (2.5 mmol) was added and the resultant mixture was refluxed for 6 days. After mixing the reactants, the clear solution turned to green suspension, which gradually thickened and darkened during the reaction time. The dark green powder product was then filtered off and washed with cold methanol and diethyl ether and dried under an infrared lamp.

[Cu_2_(*μ*-HL^2^)_2_(*μ*-Cl)_2_(HL^2^)_2_Cl_2_]·4H_2_O (**2**): Yield: 57% (wrt copper), Anal. Calc. for Cu_2_Cl_4_C_44_H_52_N_20_O_8_ (Mr = 1257.9): C, 42.0; H, 4.2; N, 22.3; Cu, 10.1. Found: C, 41.5; H, 3.9; N, 22.2; Cu 9.7%. Λ_M_ (DMF solution, S cm^2^ mol^−1^): 49.2. FTIR (Nujol, cm^−1^): 279 m (Cu–N), 315 s (Cu–Cl). FTIR (ATR, cm^−1^): 1536 m, 1592 m (C=C), 1639 s (C=N), 2920 m (C–H)_al_, 3057 m (C−H)_ar_, 3306 m (N–H). *λ*_max⁡_ (solid state, nm): 637. *λ*_max⁡_ (10^−3^ M DMF solution, nm)/*ε* (M^−1^ cm^−1^): 649/95. ESI + MS (m/z): 236, 148, 230, 293, 521, 556, 654.

In an effort to obtain single crystals of the studied complexes suitable for single crystal X-ray analysis, the dark green powder products were subjected to varied crystallization attempts, such as recrystallizations from DMF or DMSO, diffusions of diethyl ether/acetone/methanol into DMF solutions and gel crystallization from tetramethoxysilane according to [15]. Unfortunately, none of these crystallization efforts were successful and the products were at best obtained in the microcrystalline forms. However, on the basis of the results following from other techniques as well as the similarity of the presented complexes with those formerly crystallographically determined we believe that there are no doubts about the composition and stereochemistry of the studied complexes. 

## 3. Results and Discussion

### 3.1. General Characteristics

**Figure 2:**
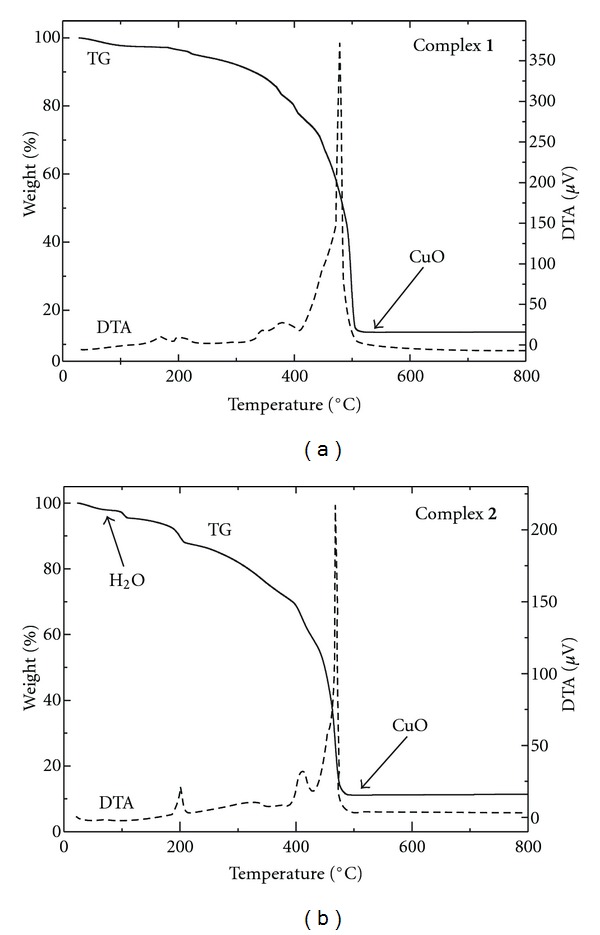
TG and DTA curves showing the thermal behaviour of complex **1** (a) and **2** (b).

The presented Cu(II) complexes [Cu_2_(*μ*_2_-HL^1^)_4_Cl_2_]Cl_2_ (**1**) and [Cu_2_(*μ*-HL^2^)_2_(*μ*-Cl)_2_(HL^2^)_2_Cl_2_]·4H_2_O (**2**) were obtained as powder products of the reactions of copper(II) chloride with the corresponding ligands (Figure 1). The differences in the synthetic procedures used lead to diverse structural types of the compounds, that is, with the bridging moieties {Cu(*μ*-HL^*n*^)_4_Cu} and {Cu(*μ*-HL^*n*^)_2_(*μ*-Cl)_2_Cu} in **1**, and **2**, respectively. It should be pointed out that the two synthetic patterns were used for both ligands, however; the obtained solids could not be characterized as magnetically pure products. The complexes were found to be well soluble in dimethyl sulfoxide (DMSO) and *N, N*′*-*dimethylformamide (DMF) and otherwise insoluble in other common organic solvents (acetone, alcohols). Various attempts to crystallize the compounds were performed to obtain single crystals of the complexes suitable for single crystal X-ray analysis (recrystallization from DMF or DMSO, diffusion of diethyl ether/acetone/methanol into DMF solutions, and gel crystallization from tetramethoxysilane according to [15]); however, still the compounds were obtained as polycrystalline solids. The molar conductivity values measured in the 10^−3^ M DMF solutions showed the complex **1** to be a 1 : 2 electrolyte (131.3 S cm^2^ mol^−1^) and the complex **2** as a nonelectrolyte (49.2 S cm^2^ mol^−1^) [16]. The simultaneous thermogravimetric (TG) and differential thermal analysis (DTA) study of **1** confirmed the complex to be nonsolvated, and the study of **2 **proved the presence of four water molecules of crystallization. The final products of thermal decomposition (Figure 2) were calculated to be CuO (complex **1**: calcd./found 14.1/13.6%; complex **2**: the loss of 4 H_2_O molecules; small *endo*-effect at 38.1°C, calcd./found 5.7/6.5%; CuO, calcd./found 12.6/11.7%).

### 3.2. Spectral Characterisations

The characteristic IR bands of the corresponding organic molecules (HL^*n*^) were observed in the IR spectra of **1** and** 2**. The most intensive **ν**(C=N)_aromatic_ vibration in the spectra of the complexes was detected at 1642 and 1639 cm^−1^ in **1** and **2**, respectively. The vibration was shifted by ca 20 cm^−1^ with respect to the spectra of free organic molecules, thus suggesting on changes in the vicinity of heterocyclic nitrogens, most likely connected with coordination to copper. In the region of the wave numbers higher than 2900 cm^−1^, the middle intensive maxima of the *ν*(C−H)_aromatic_ stretching vibrations were detected between 3057 and 3124 cm^−1^, while the peaks connected with the **ν**(C−H)_aliphatic_ were found at 2920–2933 cm^−1^. The spectra of the complexes exhibited the peaks attributable to **ν**(N−H) at 3271 and 3306 cm^−1^ for **1** and **2**, respectively. Regarding the far-IR region, the new bands, as compared to the spectra of uncoordinated organic compounds, observed at 278 (in **1**) and 279 (in **2**) cm^−1^ can be attributed to the **ν**(Cu−N) vibrations thus supporting the conclusions about coordination of the organic molecules to the central ion. The prominent **ν**(Cu−Cl) vibration was observed at 331 cm^−1^ in the spectrum of **1** and at 315 cm^−1^ for **2**, thus agreeing with the suggested bridging mode of the chlorides in **2**, because the bridging stretching **ν**(Cu−Cl) vibrations generally appear lower than the terminal ones [17].

**Figure 3:**
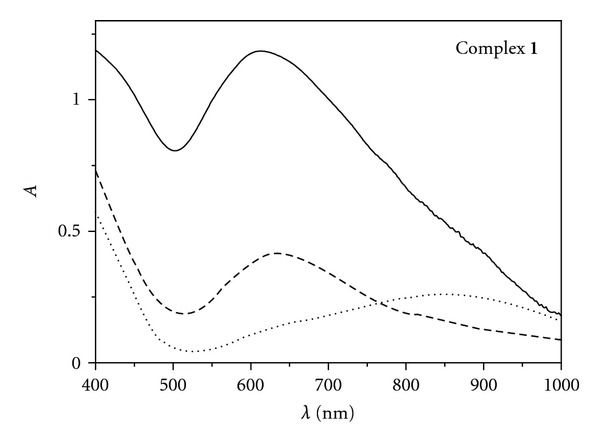
Diffuse-reflectance (solid line) and electronic (10^−3^ DMF solution) spectra of complex **1**; dashed line: freshly made solution, dotted line: after 24 h.

The diffuse reflectance and UV-Vis spectra (10^−3^ DMF and DMSO solutions) of the presented Cu(II) complexes were measured in the 200–1000 nm region. The spectra obtained in the solid state showed only one maximum corresponding to the *d–d* transition typical of the Cu(II) complexes [18]. The maxima were observed at 611 and 637 nm in the cases of **1** and **2**, respectively. This fact indirectly suggested a different coordination environment around the metal centre in **1** and **2**. The spectra of the fresh 10^−3^ DMF (or analogically DMSO) solutions (as well as of 1–3 h old solutions) of the complexes exhibited a qualitatively similar course, still the maxima were shifted to higher wavelengths by 25 and 12 nm for **1** and **2**, respectively. The calculated values of molar absorption coefficients equalled 97 (**1**) and 95 (**2**) M^−1^ cm^−1^ and correspond to the *d–d* transitions (Table 1). The solvent effect on the coordination environment of copper was clearly observable after 24 h standing of the DMF solutions, when the changes in the spectra of **1** and **2** were very significant. The intensity of the band around 640 nm decreased and a new broad peak at ca. 850 nm was observed, whose values of molar absorption coefficients also corresponded to the *d–d* transitions (~200 M^−1^ cm^−1^). The fact that the complexes partially and gradually decompose in the DMF solution could be related to the somewhat higher values of molar conductivity, especially in the case of **2**. The comparison of the diffuse reflectance and UV-Vis spectra of complex **1** are shown in Figure 3. The behaviour of the complexes in the DMSO solutions was analogical, only the peak shifts were less pronounced indicating a less significant effect of this solvent on the coordination environment.

**Figure 4:**
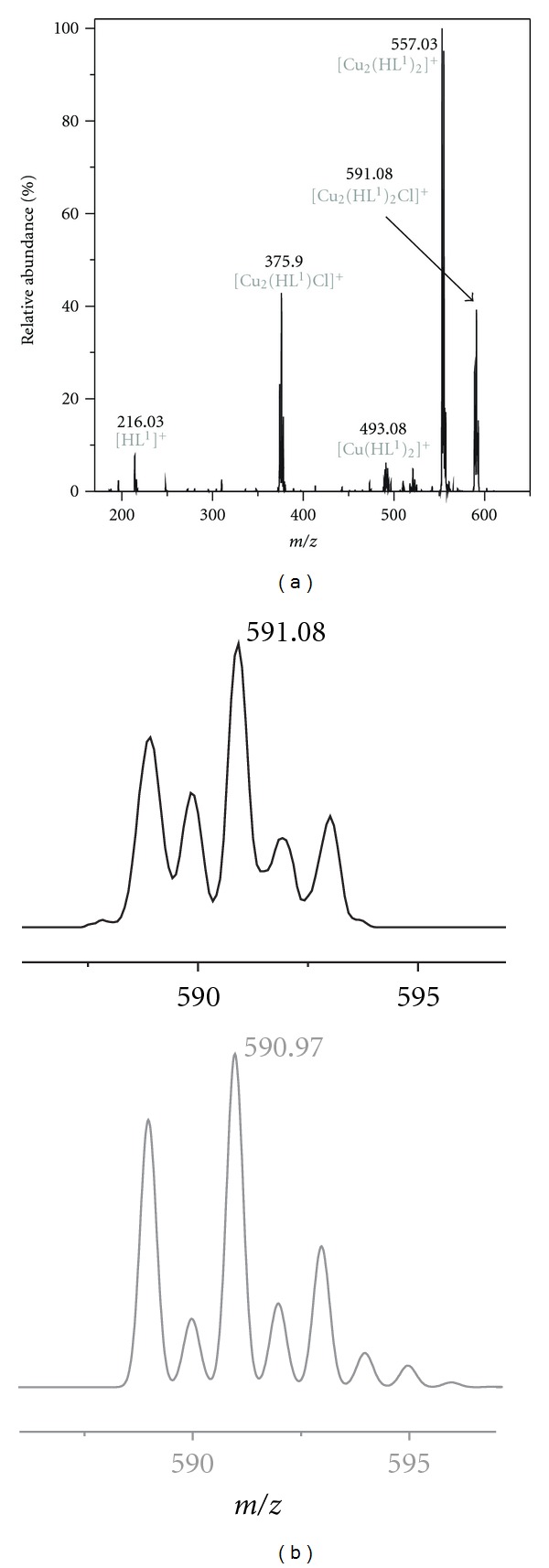
The MS^2^ spectrum showing the fragmentation of the peak 591 *m/z*, the [Cu_2_(HL^1^)_2_Cl]^+^ fragment of **1 **(left); the comparison of the measured (up) and calculated (down, in grey) isotope pattern of 591 *m/z*.

ESI+ mass spectrometry unambiguously confirmed the presence of the organic ligands (HL^1^ and HL^2^) in the complexes **1** and **2**, as the spectra revealed prominent peaks at 216 and 230 *m/z*, respectively. Then, the fragmentation of the organic molecules was also observed, as in both spectra the peaks at 136 *m/z* (adenine) and 148 *m/z* (C_5_H_4_N_5_=CH_2_; HL^1*x*^) were assigned. The molecular peak of neither complex cation of **1** nor complex **2** was detected in the mass spectra. The peaks in the ESI+ mass spectra of **1**at 278, 493, 557 and 591 *m/z* may be assigned to [Cu(HL^1^)]^+^, [Cu(HL^1^)_2_]^+^, [Cu_2_(HL^1^)_2_]^+^, and [Cu_2_(HL^1^)_2_Cl]^+^. Additionally, a dicopper particle involving a fragmented organic ligand was detected at 527 *m/z*; [Cu_2_(HL^1*x*^)_2_Cl_3_]^+^. A dicopper fragment was also observed in the negative mode spectrum, that is, [Cu_2_(L^1^)Cl_2_]^−^. Similarly, fragmentation was detected also in the spectrum of **2**. The peaks at 292, 521, 556 and 654 *m/z* may be related to the fragments [Cu(HL^2^)]^+^, [Cu(HL^2^)_2_]^+^, [Cu(HL^2^)_2_Cl]^+^, and [Cu_2_(HL^2^)_2_Cl]^+^, respectively. Another dicopper fragment was identified in the spectrum measured in the negative mode, that is, [Cu_2_(L^2^)Cl_2_]^−^at 426 *m/z*. The fragmentation of the peak at 591 *m/z*, the fragment [Cu_2_(HL^1^)_2_Cl]^+^ of complex **1,** is shown in Figure 4.

### 3.3. Magnetic Properties

**Figure 5:**
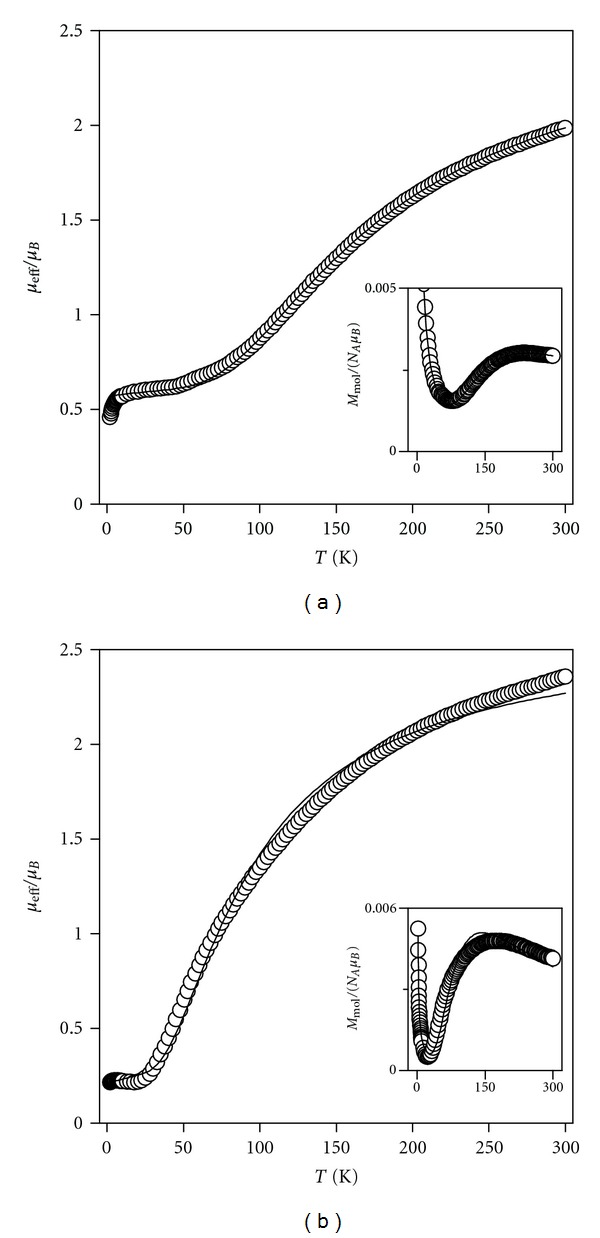
The magnetic data for complexes **1 **(a) and **2 **(b): the temperature dependence of the effective magnetic moment and molar magnetization measured at *B* = 1 T (inset). Empty circles: experimental data, full lines: calculated data using (1) and *J* = −290 cm^−1^, *g* = 2.03 and *x*_PI_ = 5.2% for **1** and *J* = −162 cm^−1^, *g* = 2.05, and *x*_PI_ = 0.7% for **2**.

The structural parameters of the presented compounds have been proposed particularly based on the data resulting from the magnetochemical characterizations. The temperature dependence of magnetic susceptibility for both compounds has the typical course for the dimers with strong antiferromagnetic coupling between the copper centres characterized by maxima at *M*_mol_ versus *T* curves, *T*_max⁡_ = 235, and 175 K for **1** and **2**, respectively (Figure 5). The magnetic data were thus analyzed with the spin Hamiltonian describing the magnetic behaviour of antiferromagnetic coupled dimers with a small amount of the monomeric paramagnetic impurity [19]:

(1) $$\begin{array}{c} \hat{H}=-J\left({\vec{S}}_{A}\cdot {\vec{S}}_{B}\right)+\mu_{\text{B}}Bg\left({\hat{S}}_{z,A}+{\hat{S}}_{z,B}\right), \end{array}$$

where the *J* parameter determines the energy gap between the singlet (*S* = 0) and triplet states (*S* = 1), resulting from the coupling of two local spins *S*_*A*_ = *S*_*B*_ = 1/2. The temperature dependence of the molar magnetization of complexes **1** and **2** were treated with simple relationship derived for such dimer [20]:

(2) Mmol=μBgNA[exp⁡((J+x)/kT)−exp⁡((J−x)/kT)][1+exp⁡((J+x)/kT)+exp⁡(J/kT)+exp⁡((J−x)/kT)],

where *x* = *μ*_B_g*B*. The total magnetization of the powder sample was calculated as the sum of the contributions of a dimer and monomeric paramagnetic impurity, *M*_mol_ = (1 − *x*_PI_)*M*_dimer_ + 2*x*_PI_ *M*_mono_. The best-fitted parameters of **1** were *J* = −290 cm^−1^, *g* = 2.03, and *x*_PI_ = 5.2% (Table 1, Figure 5). The resulting *J* value for **1** is comparable to those [*J* = (−285)–(−329) cm^−1^] reported for the Cu(II) dimers with four NCN bridges, for which the X-ray structures have been determined, for example, [Cu_2_(*μ*-AzabH)_4_Cl_2_]Cl_2_·3CH_3_OH [21], [Cu_2_(*μ*-AdeH)_4_Cl_2_]Cl_2_·6H_2_O [22, 23], [Cu_2_(*μ*-AdeH)_4_(H_2_O)_2_](ClO_4_)_4_·2H_2_O [23, 24], and [Cu_2_(*μ*-4ClLH^*x*^)_4_(ClO_4_)_2_](ClO_4_)_2_·2EtOH·H_2_O [8]; AzabH = 4-azabenzimidazole, AdeH = adenine, 4ClLH^x^ = N6-(4-chlorobenzyl)adenine. The experimental data of **2** were fitted with *J* = −162 cm^−1^, *g* = 2.05, and *x*_PI_ = 0.7% (Table 1, Figure 5), which means that the antiferromagnetic exchange for **2** was found to be substantially smaller than that for **1**. Therefore, a different exchange pathway, that is, bridging, between the Cu(II) centres is present. It has already been established that the coupling in Cu(II) dimers bridged by only chlorido bridges is generally very weak [25], therefore this cannot be the case in **2**. A similar value of *J* (–139 cm^−1^) as in **2** was found for the dimeric complex [Cu_2_(*μ*-Nphtd)_2_(*μ*-Cl)_2_Cl_2_] (Nphtd = 1,8-naphthyridine), where the Cu(II) atoms are bridged by two NCN bridges and two chlorides, that is, the {Cu(*μ*-HL^*n*^)_2_(*μ*-Cl)_2_Cu} moiety is present, which was determined by single crystal X-ray analysis [26, 27].

**Figure 6:**
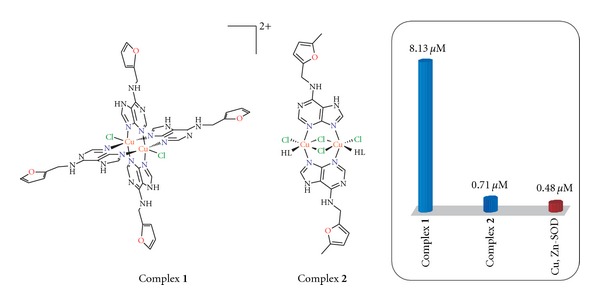
Proposed structures of the presented complexes **1** and **2** with their *in vitro* SOD-mimic activity (expressed as the IC_50_ values).

Based on the above analytical methods and literature research, for complex **1**, we propose the 1 : 2 ionic composition of the complex cation [Cu_2_(*μ*-LH^1^)_4_Cl_2_]^2+^, with four *μ*-N3,N9 bridging kinetin molecules and two terminal chlorides coordinated to Cu(II), whose charge is compensated by two chloride anions. The proposed structure for **2** involves the dimeric core {Cu(*μ*-HL^*n*^)_2_(*μ*-Cl)_2_Cu} with the copper centres bridged by two *μ*-N3,N9 HL^2^ molecules and two chlorides, with one terminal N9-coordinated HL^2^ and one chloride bonded to each Cu(II) (Figure 6), as was analogically reported previously for [Cu_2_(*μ*-*n*ClHL^*x*^)_2_(*μ*-Cl)_2_(*n*ClLH^*x*^)_2_Cl_2_]·2H_2_O (*n*ClHL^*x*^ = N6-(*n*-chlorobenzyl)adenine; *n* = 2, 3) [28, 29].

### 3.4. Evaluation of SOD-Mimic Activity

The antiradical activity was evaluated for the presented compounds by the *in vitro* SOD-mimic test performed by a slightly modified indirect chemical method [13]. The results for the tested Cu(II) complexes, as compared with native bovine Cu, Zn-SOD (IC_50_ = 0.48 *μ*M, [13]), indicated that these compounds could be considered good SOD-mimics (Figure 6) because the IC_50_ values equalled 8.13 (for **1**) and 0.71 *μ*M (**2**). The more potent compound **2** exhibited the activity of the same order of magnitude as Cu, Zn-SOD as well as the most active Cu(II) complexes with adenine derived N-donor ligands (IC_50_ = 0.253–1.250 *μ*M) prepared previously in our laboratory and having additionally strong *in vivo* antidiabetic activity [9]. Direct comparisons of SOD-mimic activity results of complexes **1** and **2** with other Cu(II) complexes reported before are somewhat complicated by diverse methodology used for the generation of superoxide. What can only be compared is the ratio of the activity of the studied compound and the native SOD determined by the same procedure. Using this logic, the activity of the herein presented complex **1** (17 × higher IC_50_ than native bovine Cu, Zn-SOD) is comparable or even better than various reported Cu(II) complexes exhibiting pharmacological properties connected to the dismutation of the superoxide radical. For example, the Cu(II) complex used as a SOD mimic in veterinary practice [Cu_2_(indo)_4_(DMSO)_2_] (indo = indomethacin) was in the testing found to have ca 6 × higher IC_50_ than SOD [30], while the Cu(II) complexes possessing antirheumatic activity involving D-penicillamine (200 × higher IC_50_ than SOD) or 2-merkaptopropionylglycine (150 × higher IC_50_ than SOD) [31] can be in this comparison understood as worse SOD-mimics than complex **1**.

From the general point of view of the SOD-mimic active types of Cu(II) complexes, it has already been shown that the dimeric compounds tend to be more active than the mononuclear ones. It was previously proven for example for the dimeric and monomeric Cu(II) complexes with tripodal polypyridyl-amine ligands, where the monomeric complexes (IC_50_ = 5.02–140.0 *μ*M) were significantly less antioxidant active than the dimeric ones (IC_50_ = 0.54–0.76 *μ*M) [32]. It is generally explained by the possible cooperation of both Cu(II) centres in electron transfer and binding of free radicals. This finding can be also demonstrated by data recently acquired in our laboratory concerning the SOD-mimic activity of Cu(II) complexes involving various derivatives of the title molecule kinetin. The mononuclear complex [Cu(H_2_O)_2_(L^*a*^)_2_(phen)] exhibited a very low antioxidant effect (IC_50_ = 189.6 *μ*M). The dimeric complex [Cu_2_(*μ*_2_-Ac)_4_(HL^*b*^)_2_] bridged by four acetate groups was more active (57.4 *μ*M) [33], however, still not reaching the activity exhibited by the herein presented compounds; HL^*a*^ = 2-chloro-N6-furfuryladenine, HL^*b*^ = 2-chloro-N6-furfuryl-9-isopropyladenine. 

The active site of Cu, Zn-SOD has the Cu(II) atom coordinated by four imidazole moieties from the histidine molecules and by one water molecule in the distorted square pyramid, with the CuN_4_O chromophore [34]. The copper and zinc metal centres are bridged by the deprotonated N-donor imidazole moiety from one of the histidine molecules. In complex **1**, each Cu(II) atom is also pentacoordinated by four nitrogen atoms from the N-donor bridging organic ligands (HL^1^) and by one chlorido ligand, thus forming the CuN_4_Cl chromophore. On the other hand, in complex **2**, the Cu(II) atoms are octahedrally coordinated by three nitrogen atoms from the bridging and terminal organic ligands (HL^2^), two bridging and one terminal chlorido ligands in the CuN_3_Cl_3_ donor set. There are two aspects from the structural point of view that could help to explain the higher SOD-mimic activity of the herein presented complexes as compared to the previously reported Cu(II) complexes involving kinetin derivatives [33]. Firstly, the higher activity of **1** and **2** might be connected with the type of bridging between the copper centres, which is different from the previously described dimeric complexes (acetato bridges). Therefore, the presence of the bridging bidentate N-donor HL^*n*^ molecules in **1** and **2** (similarly to bridging imidazole in native Cu, Zn-SOD) might help to mimic, to some extent, the structure of the active centre of SOD-enzyme and thus be able to, just like the bridging imidazole, participate in the mechanism of dismutation. Secondly, besides the type of bridging; what clearly also influences the antioxidant activity of a Cu(II) complex is the accessibility of the metal ion for superoxide [32]. This might be the reason why the previously reported dimeric Cu(II) chlorido complexes of N6-benzyladenines are more than twenty times more active than the analogical perchlorate compounds both involving the same type of bridging bidentate N-donor ligands [8, 9]; for example, [Cu_2_(*μ*_2_-4MeOHL^*x*^)_4_Cl_2_]Cl_2_·2H_2_O (IC_50_ = 0.687 *μ*M) contrary to [Cu_2_(*μ*_2_-4MeOHL^*x*^)_4_(ClO_4_)_2_](ClO_4_)_2_ (IC_50_ = 14.41 *μ*M); 4MeOHL^*x*^ = N6-(4-methoxybenzyl)adenine. The bulky ClO_4_^−^ anionic ligand makes it more difficult for superoxide to access the metal centre and to substitute this ligand on copper in the suggested first phase of the dismutation process [8]. The same logic might be applied when explaining the different activity of the herein presented compounds **1** and **2**. The compound **1** is more than ten times less active than **2**, because the copper centre accessibility could be lower in the dimeric core of **1** due to the presence of four bulky N3,N9-bridging kinetin molecules, while the bridging in **2** is formed by two N3,N9-bridging HL^2^ molecules and two bridging chlorides with higher bond length and angle flexibility. The higher activity of **2** with the {Cu(*μ*-HL^*n*^)_2_(*μ*-Cl)_2_Cu} bridging moiety is in agreement with the finding that the most active Cu(II) compound involving an adenine derivative has the composition [Cu_2_(*μ*-HL^*a*^)_2_(*μ*-Cl)_2_Cl_2_] [9] with the same bridging unit as **2**.

## 4. Conclusion

In conclusion, this work presents synthesis and characterization of two Cu(II) complexes of the compositions [Cu_2_(*μ*_2_-HL^1^)_4_Cl_2_]Cl_2_ (**1**) and [Cu_2_(*μ*-HL^2^)_2_(*μ*-Cl)_2_(HL^2^)_2_Cl_2_]·4H_2_O (**2**), where HL^1^ stands for kinetin and HL^2^ for methyl-derived kinetin (N6-(5-methylfurfuryl)adenine). The complexes were evaluated for their antiradical activity, which resulted in the IC_50_ values equalling 8.13 *μ*M (complex **1**) and 0.71 *μ*M (**2**), therefore the presented compounds might be characterized as auspicious SOD-mimics.

## Figures and Tables

**Table 1:** Magnetic and electronic spectral data for complexes **1** and **2**.

Compound	*J*[cm^−1^]	*g*	Diffuse-reflectance spectra [nm]	UV-Vis spectra (fresh DMF solution) [nm] (*ε* [M^−1^/cm])	UV-Vis spectra (24 h old DMF solution) [nm] (*ε* [M^−1^/cm])
**1**	−290	2.03	611	636 (97)	640^a^, 860 (195)
**2**	−162	2.05	637	649 (95)	652^a^, 840 (150)

^a^Detected as a shoulder of the band at higher wavelength.
